# Bridging the gap: The experiences and needs of people with spinal cord injury when transitioning out of inpatient rehabilitation

**DOI:** 10.4102/sajp.v82i1.2260

**Published:** 2026-04-13

**Authors:** Cleopatra Floudiotis, Sonti I. Pilusa

**Affiliations:** 1 Department of Physiotherapy, Faculty of Health Sciences, University of the Witwatersrand, Johannesburg, South Africa

**Keywords:** spinal cord injury, rehabilitation, experience, transition, environmental factors, personal factors, needs, specialised care

## Abstract

**Background:**

The rehabilitation experience can influence the transition back to home and community life. With impending National Health Insurance reform and limited rehabilitation guidelines in South Africa, there is a need to understand the current state of rehabilitation services. To the authors’ knowledge, no other South African study has explored inpatient rehabilitation experiences and their influence on the readiness to transition home in spinal cord injury (SCI).

**Objectives:**

To explore the experiences of inpatient rehabilitation among people with SCI, to examine how the experience influenced their transition out of inpatient rehabilitation, and to identify the needs of people with SCI when transitioning out of inpatient rehabilitation.

**Method:**

A phenomenological, qualitative design was used. Semi-structured interviews were conducted with 11 patients with SCI in their final week of inpatient rehabilitation from October 2023 to February 2024. The interview guide was developed from similar studies in New Zealand. Interviews were transcribed verbatim and inductively analysed to identify themes.

**Results:**

Three themes emerged: overall rehabilitation experience, factors influencing the experience and transition readiness, and needs impacting the transition to home. Participants reported a positive experience overall, although some negative elements were noted. Key needs for a smoother transition included bridging the gaps in the approach to care, more realistic home environment simulations and individualised psychological support.

**Conclusion:**

Rehabilitation experiences highlight the need for a specialised, patient-centred approach to SCI care. Addressing unmet needs can enhance preparedness for reintegration, especially in contexts without a structured SCI model of care.

**Clinical implications:**

Ongoing professional development, improved rehabilitation environments and structured psychological support during rehabilitation are needed.

## Introduction

Rehabilitation following a spinal cord injury (SCI) addresses the resulting physical impairments and functional limitations, which can lead to poor quality of life (QoL) (Cheng et al. 2017) and an increased burden of care (Turner-Stokes et al. 2016). According to the World Health Organization (WHO), rehabilitation should be guided by the categories highlighted in the International Classification of Functioning, Health and Disability (ICF), namely health condition, body functions, activity limitations, participation restrictions, environmental factors and personal factors. The use of these categories as guidelines for care by an interdisciplinary team should aimed at optimising functioning within the person’s environment while keeping the individual’s goals, preferences and circumstances at the centre, with the ultimate goal of enabling people with SCI to return to productive life and to self-manage as far as possible (Du Plessis et al. 2018; Pefile, Mothabeng & Naidoo 2019).

The WHO has put forward a global action initiative to strengthen rehabilitation care by 2030 (WHO 2017). This initiative states that rehabilitation care should be accessible and available at all levels of care to all people with disabilities to allow them to participate in home and community life (WHO 2017). Currently, there is no one SCI care model that is universally used (Ho et al. 2021). In addition, most of the evidence on SCI rehabilitation has been conducted in high-income countries, such as Australia, the United States and Canada (Ho et al. 2021). In low- and middle-income countries such as South Africa, however, contextual evidence on SCI rehabilitation is limited.

In South Africa, rehabilitation care for people with disabilities, including SCI, is not prioritised and needs strengthening (Morris et al. 2021). With the impending reform to a National Health Insurance (NHI) system and limited guidelines on rehabilitation in South Africa, there is a need to understand the current state of rehabilitation services (Louw et al. 2023). There are myriad challenges facing rehabilitation in South Africa, including limited services at primary healthcare levels, inadequately skilled healthcare workers, a lack of specialised services for long-term care and inadequate referral systems (Louw et al. 2023).

These challenges can impact long-term care outcomes for people with SCI. For example, Madasa found that the mortality rate 4 years after injury was 24% in patients with traumatic SCI (Madasa et al. 2020). People with SCI also experience secondary health complications, including pressure sores, urinary tract infections and pain, which increase readmission rates and negatively impact QoL (Kashif et al. 2019; Madasa et al. 2020; Mashola et al. 2019). In addition to the health complications, SCI can also limit participation in community activities (Kashif et al. 2019). These activities include sustaining employment and education, and participating in social activities, the lack of which can lead to financial insecurity, isolation (Tsai et al. 2017), and poor QoL (Smith, Sakakibara & Miller 2014). Although the national policy on rehabilitation recognises the importance of community reintegration, most rehabilitation services in South Africa do not progress beyond the achievement of basic mobility and self-care (Nizeyimana, Louw & Joseph 2025). Combined with the environmental, psychological and social barriers that people with SCI face, this situation emphasises the need to understand the experiences, and more importantly, the gaps in existing inpatient rehabilitation services.

The objectives of our study were to explore the experiences of inpatient rehabilitation among people with SCI, to explore how the experience influenced their transition out of inpatient rehabilitation, and to identify the needs of people with SCI when transitioning out of inpatient rehabilitation. To the authors’ knowledge, there is no other study in South Africa exploring the experiences of inpatient rehabilitation and their influence on the readiness to transition home in SCI.

## Research methods and design

### Study design

A qualitative, phenomenological design was used to explore the experiences of rehabilitation in people with SCI.

### Study setting

Our study was conducted at a private rehabilitation hospital offering multidisciplinary care for SCI, stroke, orthopaedic polytrauma and acquired brain injury. In the spinal unit, patients typically receive individual and group physiotherapy and occupational therapy. The average length of stay for SCI patients is 6–12 weeks.

Discharge preparation includes weekend home trials. Most patients are funded through private medical aid or state compensation funds (e.g. Workmen’s Compensation or Road Accident Fund), with some self-funding their care.

### Study population

Patients with SCI, regardless of aetiology or level of lesion, who were over the age of 18, who were within their first week of discharge, and who had received inpatient rehabilitation at the spinal unit for at least 6 weeks were recruited by the first author, with the assistance of an employee at the rehabilitation hospital. We excluded patients if they were admitted with any secondary complications, such as pressure sores, or had sustained any other injury or event which resulted in severe cognitive fallout.

A physiotherapist employed at the hospital screened and identified patients who met the criteria and facilitated introductions between the authors and potential participants. All patients approached during the data collection period agreed to participate in our study. Eleven participants were recruited. Although additional recruitment was possible, the number of participants was partly influenced by the natural flow of eligible discharges during our study period.

Data saturation was assessed retrospectively during the analysis. Although a formal saturation table was not used, the first author kept an Excel spreadsheet documenting emerging codes, meaning categories and illustrative quotations, thus monitoring for meaning saturation (Morse 1995; Saunders et al. 2018). As the analysis progressed, it became evident that interviews produced repeating patterns rather than new conceptual material.

The first two themes stabilised earlier in the process, while the final theme reached saturation later. By the final interviews, all themes and subthemes had been fully developed. The last interviews confirmed and deepened existing insights rather than generating new ones. In keeping with phenomenological research standards, in which sample sizes typically range from five to 25 participants and are driven by depth rather than volume (Creswell 1998; Guest, Bunce & Johnson 2006), the sample of 11 provided detailed accounts across all interview domains. While further interviews may have added further depth or additional nuance within existing themes, they were unlikely to generate new conceptual material. The final sample was therefore considered sufficient to address the aim of our study.

### Data collection

Semi-structured interviews were conducted by using an interview guide developed by the authors. The interview guide was informed by interview guides from similar studies conducted in New Zealand (Bourke et al. 2014; Nunnerley & Dean 2013) and clinical experience. A pilot study was conducted to test the flow and clarity of the questions, leading to refinements of the guide. The interview guide had two parts: section A was a questionnaire used to gather demographic information (see Table 1) and was based on demographic information gathered by another South African study (Pilusa, Myezwa & Potterton 2021). Section B included open-ended questions with probes, which were selected based on the ICF framework (WHO 2002). The probes assisted in categorising the ‘hospital environment’ into factors such as physical ward and therapy areas, staff involvement and psychosocial support. The final questions in section B were related to the needs of people with SCI while in rehabilitation and in the transition to home.

**TABLE 1:** Demographic profile (*N* = 11).

Variable	*n*	%	Mean	s.d.	Range
**Age (years)**	-	-	45.7	19.46	18–68
**Gender**					
Men	9	81.8	-	-	-
Women	2	18.2	-	-	-
**Race**					
Black people	5	45.4	-	-	-
White people	4	36.4	-	-	-
Coloured people	2	18.2	-	-	-
**Marital status**					
Married or partnership	6	54.4	-	-	-
Single	4	36.4	-	-	-
Widowed	1	9.1	-	-	-
**Employment**					
Before injury					
Employed	8	72.7	-	-	-
School-going	1	9.1	-	-	-
Retired	1	9.1	-	-	-
Medically boarded	1	9.1	-	-	-
Employed with temporary disability leave	0	0.0	-	-	-
On discharge					
Employed	4	36.4	-	-	-
School-going	1	9.0	-	-	-
Retired	1	9.1	-	-	-
Medically boarded	1	9.1	-	-	-
Employed with temporary disability leave	4	36.4	-	-	-
**Place of residence**					
Before injury					
Family home or own home	11	100.0	-	-	-
Work rehabilitation facility	0	0.0	-	-	-
Temporary work accommodation	0	0.0	-	-	-
On discharge					
Family home or own home	8	72.7	-	-	-
Work rehabilitation facility	2	18.2	-	-	-
Temporary work accommodation	1	9.1	-	-	-

s.d., standard deviation.

A pilot interview was conducted telephonically with one participant by the first author. The participant had consented to participate but was discharged earlier than anticipated, thus necessitating a telephonic interview.

The pilot was used to assess the clarity and comprehensibility of questions, the flow and sequencing of the interview guide, the duration of the interview, and the relevance of the questions to our study aims. It also provided an opportunity for the first author to refine interviewing skills. The pilot interview was audio-recorded, transcribed, and reviewed by both authors. This process informed several refinements to the interview guide, including adding additional probing questions to elicit richer descriptions. The pilot also enabled testing of the transcription process. As the content of the pilot interview was relevant and met all inclusion criteria, it was included in the final dataset.

All subsequent interviews were conducted by the authors in person at the rehabilitation hospital within the final week of each participant’s rehabilitation stay. English interviews were conducted by the first author, and Zulu interviews were conducted by the second. The interviews were audio-recorded and transcribed verbatim. Interviews conducted in Zulu were translated into English by an external translator for data analysis.

Each participant was given a pseudonym to ensure anonymity. The average length of the interviews was 30–45 min, and they were conducted from October 2023 to February 2024. Once data saturation was reached, the interview process was concluded.

### Data analysis

The data were manually analysed by using a thematic approach, followed by collaborative discussions between the authors to refine codes and categories. The process included familiarisation with the transcripts, initial inductive and deductive coding, grouping codes into categories, and refining these into three overarching themes. A detailed outline of the coding process is provided in Appendix 1 Table 1-A1.

Trustworthiness was ensured by using established strategies (Krefting 1991; Nowell et al. 2017). Credibility was supported by applying clear inclusion criteria and engaging in regular discussions about data collection and analysis. Method triangulation involved both inductively coding, allowing themes to emerge from participants’ narratives, and deductive coding, using concepts from similar studies (Bourke et al. 2014; Nunnerley & Dean 2013) and the ICF framework. This approach also enhanced credibility by grounding themes in participant experiences while reducing bias from prior assumptions. Dependability was enhanced by re-analysing a portion of the data for consistency. Transferability was addressed by including participants’ demographic details, and confirmability was supported through audio recording of all interviews.

### Reflexivity

As a clinician experienced in neurological rehabilitation across acute, subacute and outpatient settings since 2017, I have observed inadequacies in SCI rehabilitation services in preparing patients for life at home. This realisation has led me to question whether hospital-based rehabilitation is the most effective approach for facilitating patients’ transitions to their home, community, and new way of life.

As a white female physiotherapist, my background inevitably shapes how I engage with participants and interpret their experiences. My professional background may lead me to focus on the clinical elements of rehabilitation, while my social identity and personal experiences may influence how I perceive and relate to participants, especially across cultural, racial or gender differences. I used reflexive journaling and peer debriefing to mitigate these influences through challenging assumptions, interpretations and potential biases.

### Ethical considerations

The recruitment and data collection process was started only once permission was granted by the rehabilitation hospital group, and once ethical clearance was given by the Human Research Ethics Committee of the University of the Witwatersrand (reference number: M2111137). We explained our study to the participants in English or Zulu, and they all gave written or verbal informed consent to be interviewed and to be audio-recorded.

## Results

The sample in our study included 11 patients with SCI. Most of the participants were men (81.8%) and had traumatic injuries (72.7%). Most of the participants’ injuries were thoracic (54.5%) and incomplete (81.8%). Table 1 and Table 2 show the demographic and SCI profile of the participants.

**TABLE 2:** Spinal cord injury profile.

Variable	*n*	%	Mean	s.d.	Range
**Cause of injury**					
Trauma	8	72.7	-	-	-
Non-trauma	3	27.3	-	-	-
**Level of lesion**					
Cervical	4	34.4	-	-	-
Thoracic	6	54.5	-	-	-
Lumbar	1	9.2	-	-	-
**Completeness of injury**					
Complete	2	18.2	-	-	-
Incomplete	9	81.8	-	-	-
Length of stay (weeks)	-	-	8.45	1.44	6–11

s.d., standard deviation.

The themes which emerged were: ‘overall rehabilitation experience’, ‘factors influencing rehabilitation experience and their influence on readiness to transition out of rehab’, and ‘needs of people with SCI when transitioning out of inpatient rehabilitation’. Table 3 shows the themes, categories and subcategories that emerged from the data analysis. Appendix 1 Table 1-A2 shows the distribution of participant responses across themes and subthemes identified in the qualitative analysis (*n* = 11).

**TABLE 3:** Themes, categories and subcategories.

Themes	Categories	Subcategories
Overall rehabilitation experience	-	Overall experience
		Hospital services received
Factors influencing rehabilitation experience and their influence on readiness to transition out of rehabilitation	Environmental	Care approach: staff attitudes and knowledge, communication, and involvement in decision-making
		Physical hospital environment
		Peer support
	Personal	Emotions
		Beliefs and motivation
		Perceived readiness to go home
Needs of people with SCI when transitioning out of inpatient rehabilitation	Patient-centred care	-
	Real-life rehabilitation environment or home trials prior to discharge	-
	Enhancing psychological support	-

SCI, spinal cord injury.

### Overall rehabilitation experience

Overall, 72.2% (*n* = 8) of participants described their inpatient rehabilitation experience as positive, finding it helpful, motivating and enjoyable:

‘It’s been nice, they helped me a lot. I couldn’t do anything before I came here. I just lay there. Now I can move. I’m stronger. I can push a wheelchair. I can get on and off.’ (Michael, 18 years old, T3 incomplete)

‘Rehab, it was a good experience … and because of the rehab and everything, the people there helped me a lot, and they gave me more insight into what to expect, and just to work hard. And it paid off.’ (Joe, 25 years old, C3 incomplete)

While most feedback was positive, 27.2% (*n* = 3) of participants shared mixed views and noted minor areas of dissatisfaction:

‘It’s been good … otherwise, there are a few little things that I wasn’t happy with.’ (Jane, 66 years old, T9 complete)

‘I can say 98%, it was good, 2% it was bad … ’ (Hloni, 48 years old, C4 incomplete)

#### Hospital services received

Participants described receiving rehabilitation services from nurses, doctors, physiotherapists, occupational therapists, social workers, and psychologists, primarily delivered in two settings: the ward (nurses, doctors and psychologists) and the gym (therapists and social workers). Nine participants (81.8%) received all of the above services, while two (18.2%) reported that they did not feel that they needed psychology services.

Nurses focused on teaching and assisting with bowel and bladder management and daily activities in the ward, while doctors conducted daily rounds, monitored patients and addressed medical issues. Physiotherapists and occupational therapists provided training in wheelchair mobility, transfers, gait re-education (where appropriate) and activities of daily living (toileting, showering and dressing). Social workers offered counselling, coordinated family meetings, facilitated home visits, and liaised with employers. Psychologists provided support in trauma management, sexual counselling and managing patients’ expectations.

### Factors influencing rehabilitation experience and their influence on readiness to transition out of rehabilitation

The participants highlighted various factors influencing the rehabilitation experience and the transition out of rehab, and these were categorised into environmental and personal factors.

#### Environmental factors

The environmental factors highlighted by the participants included the care approach (staff attitudes, communication and involvement in decision-making), the physical hospital environment and peer support.

#### Care approach: Staff attitudes and knowledge, communication, and involvement in decision-making

All participants emphasised the impact of the hospital staff’s attitudes and approach to care on their overall experience. Most healthcare professionals were described as friendly, helpful and supportive:

‘The nurses that are here, they are the best. Whenever I call them, they will come to help me as soon as possible.’ (Thapelo, 21 years old, T5 incomplete)

‘They never refuse to help you, there is always a person present to help.’ (Lethabo, 65 years old, T12 incomplete)

However, 63.6% (*n* = 7) of participants had mixed experiences when it came to the care approach by health professionals, noting that a few staff members did not meet their expectations or displayed negative attitudes:

‘Some of the nurses are nice. Some of them are not so nice … If I treat you humanely, just do the same, that’s all I ask.’ (John, 68 years old, L3 incomplete)

‘I think it’s the situation of the day. You know, if they know you’ve just had a suppository and you are messed, some of them tend to avoid you … ’ (Jane, 66 years old, T9 complete)

Participants noted that some healthcare workers lacked the necessary knowledge or skills to effectively assist patients with SCI. They emphasised the importance of staff being more aware of each person’s individual needs and limitations:

‘All of them know how to transfer you, and all of them know how to put you on a commode. But according to them, they don’t know … So you’re getting the sister in charge of them saying they are trained to do transfers, and they are trained for commodes, but they say they’re not.’ (Amy, 56 years old, T8 incomplete)

‘The staff don’t quite know … Okay, so here’s your food … I can’t move either of my arms, so now what? Oh, I forgot. So those kinds of things early days were very frustrating.’ (Mark, 48 years old, C1 incomplete)

The participants highlighted how communication and their involvement in decision-making affected their rehab experience. Communication was an important element of the participants’ rehabilitation experience.

Participants expressed that communication among the healthcare workers involved in their care was helpful for continuity of care within rehabilitation, but this did not appear to have a direct impact on the transition home:

‘Even when your main people [*healthcare providers*] aren’t there, there’s enough of an understanding of where I am, where they can take over to probably an 80% idea of kind of stepping in, they can kind of continue with it instead of starting from scratch … ’ (Mark, 48 years old, C1 incomplete)

‘If he’s [*the doctor*] not gonna come tomorrow, he tells you, Dr [*the doctor*] will come and visit you … ’ (Hloni, 48 years old, C4 incomplete)

Communication about discharge plans was lacking in some cases, and this made participants feel uncertain and unprepared for their discharge:

‘It was a shock when they told me, you are leaving end of December … ’ (John, 68 years old, L3 incomplete)

‘I am asking myself why they are moving me from here. Even if we fought … but we should move on … No one explained anything to me, but you can tell from the way they treat you that there are issues that are not resolved.’ (Siya, 61 years old, C5 incomplete)

Some participants felt that healthcare professionals did not listen to them or involve them in decision-making about their medical care. This lack of involvement negatively affected their rehabilitation experience and their ability to self-manage:

‘Also, you know you’re doing the bowel regime. I had a problem with that as well. The suppository, I would not get it in time … You’re supposed to have it and then … go to the commode, go to the bathroom. That never happened. They would never bring it; there was always an excuse, and they just left you. That’s been a big issue. So, to the point that I haven’t got into a routine yet. And I am suffering from constipation.’ (Jane, 66 years old, T9 complete)

‘I am a black person, and part of my culture does not allow me to be cut in certain areas of the body … I then discussed it with my family, and we made a decision … I told the doctor about our decision, and the doctor was not happy about it … [*doctor*] was furious that we continued talking about the same issue, and I don’t want to do what the doctor wanted, even though it would help me. Yes, it helps me, but tomorrow it might not be good for me. The doctor does not know what will happen in future. So, we ended up not seeing eye to eye with the doctor.’ (Siya, 61 years old, C5 incomplete)

#### Physical hospital environment

The ward and the gym were the two areas where participants spent most of their time. Most participants (72.7%, *n* = 8) had both positive and negative comments about the physical environment. Participants found the ward environment clean and spacious, but it was also noisy:

‘The other one [*main ward*] wasn’t so good, but this one [*smaller side ward*] is [*good*]. It’s clean and it’s quiet … That one is noisy. But yeah, it’s nice here.’ (Michael, 18 years old, T3 incomplete)

‘The bottom line is, you don’t sleep properly here, ever … You’re working hard downstairs … and your body is still trying to recover. You are pretty much getting to sleep at, maybe, 10:00, 10:30, and you will be awake at about 04:00, excluding anything else that might happen in the night. And the general ward is worse.’ (Mark, 48 years old, C1 incomplete)

Some participants found that the bathrooms were difficult to access. In addition, bathroom equipment, commodes in particular, was in poor working order and sparsely available:

‘So when you get a commode, you either get a commode that’s got no footrests, so your feet are dangling … 90% don’t even have working footrests.’ (Amy, 56 years old, T8 incomplete)

‘The commode. What a disaster. They’re all broken. They don’t work. That is something that needs to be looked into. There are so many around, but yet when you want one, there’s never one available.’ (Jane, 66 years old, T9 complete)

#### Peer support

Being in an environment among other patients with SCI, with whom they could share their experiences, made 45.5% (*n* = 5) participants feel more comfortable and less isolated, while 18.2% (*n* = 2) found the presence of peers both encouraging and difficult at times. Peers with SCI were encouraging towards one another, which made participants feel supported. Being exposed to other people with varying levels of disability gave participants perspective on their injuries:

‘They [patients in the ward] keep encouraging me that I should not give up, I will be able to walk again. I am not the only one; there are many more. I am not the first person to experience this situation … ’ (Mpho, 27 years old, T11 complete)

‘Friendship I have with the people in the ward, when we are done, we talk about where we come from … how we got injured … ’ (Siya, 61 years old, C5 incomplete)

The transition to home may have been impacted by this, as some participants were aware that this would not be the case at home or in their communities:

‘I feel more comfortable here because there’s people in wheelchairs. It’s not the same like at home.’ (Michael, 18 years old, T3 incomplete)

Participants valued the encouragement and shared understanding gained from peers with SCI during rehabilitation, although some anticipated the loss of this support once they returned home.

### Personal factors

All of the participants expressed how they played a part in preparing for home. The personal factors which influenced their experiences were their emotions, beliefs and motivation, and their perceived readiness for discharge.

#### Emotions

Of the participants, 90% (*n* = 10) expressed that their emotional states throughout the care continuum affected their experience. Some had to deal with and overcome the trauma of their injuries or acute hospital experiences:

‘In the beginning, it was a little bit difficult … Because even with my injury, I also had the trauma of my mom being involved [*in the incident*] as well.’ (Joe, 25 years old, C4 incomplete)

‘Since I got injured, I get scared when travelling with a car. I sometimes worry that if I get another accident, I will be badly injured.’ (Lethabo, 65 years old, T12 incomplete)

Some explained that they felt uncertain and hopeless at the start of their rehabilitation journeys, but with encouragement and learning skills, they felt more confident:

‘I mean, in the beginning, when you get here, you’re like, lost. You don’t know like, what you’re gonna do … But afterwards your confidence comes, and you just learn.’ (Amy, 56 years old, T8 incomplete)

‘First of all, when I first got injured, when I came here, I had given up. But when I came here, they told me, “You must never give up, you are still young, you can make it.”’ (Thapelo, 21 years old, T5 incomplete)

#### Beliefs and motivation

The participants’ own beliefs and attitudes had a big impact on their experience in rehabilitation for 72.7% (*n* = 8) of participants. They expressed the need to believe that they would get better, and that having internal and external motivation was important. The participants used acceptance, spirituality and self-determination to encourage themselves. Acceptance of the current situation made the experience easier, and taking responsibility for their own rehabilitation was important in achieving goals and becoming independent:

‘I made my heart and mind understand that my situation has changed and this is my present situation.’ (Mpho, 27 years old, T11 complete)

‘Here it is up to you … It is up to you to decide if you are going to continue do the exercises … ’ (Siya, 61 years old, C5 incomplete)

#### Perceived readiness to go home

Participants had mixed emotions when it came to being discharged from the rehabilitation hospital, 54% (*n* = 6) reporting that they felt prepared and positive about going home:

‘I am happy and ready to go home and practice what they taught me here to see if I can manage.’ (Mpho, 27 years old, T11 complete)

and 18.2% (*n* = 2) felt positive, but also apprehensive:

‘I feel confident … I am a bit scared. When I go home and it’s a different life … You know I like to just get up and go and do and run … and now I can’t.’ (Jane, 66 years old, T9 complete)

Part of what made patients apprehensive about going home was reintegration into the community, and the attitudes of the people they would face:

‘People’s reactions in the township. They know me being able to walk by myself, now I … am using a wheelchair, I’m worried about what they will say. I am embarrassed about it.’ (Mpho, 27 years old, T11 complete)

‘That is something I’m not looking forward to … [*socialising upon discharge*].’ (Jane, 66 years old, T9 complete)

### Needs of people with spinal cord injury when transitioning out of inpatient rehabilitation

The experiences of the participants highlighted that there were three main care needs which influenced the transition to home.

### Patient-centered care

Firstly, 45.5% (*n* = 5) of participants highlighted the need for a more patient-centred approach to care, particularly regarding SCI-specific self-management skills. Those who were encouraged to take responsibility for their self-management in the ward reported feeling better prepared for transitioning home:

‘Once you get to the point where you can turn yourself, then they leave you to yourself. It’s your responsibility. I suppose also preparing you for when you go home.’ (Jane, 66 years old, T9 complete)

‘When I first came, there was a nurse I used to call and request her to change my nappy. She would change it and told me that I must learn to change it myself. She emphasised that I need to learn because one day I will go home, and nurses won’t be there to help me … For her to be strict with me helped me a lot.’ (Mpho, 27 years old, T11 complete)

However, in some instances, there remained a gap between the skills learnt in structured rehabilitation sessions and those practised in the ward, especially in SCI-specific self-management:

‘I didn’t go to the bathrooms. Because here the nurses are too scared to take you there. So you’ve got your OT, she teaches you everything … But in the ward, they are too scared to take you to the bathroom … and I asked the sister, and he said, “No, he’s not doing it, it’s an OT’s job.” I said, “No, the OT is there to teach me to do it. You are there to assist me to do it.” He said, “No, it’s not his job.”’ (Amy, 56 years old, T8 incomplete)

Some participants highlighted that the attitudes of rehabilitation staff can impact the experience. While healthcare professionals may perceive patient interactions as part of their routine professional duties involving numerous daily encounters, patients experience these interactions as part of deeply personal, involuntary and life-altering circumstances. This insight highlights the need for ongoing professional development and training for all rehabilitation staff on specialised SCI care and the patient-centred approach to care:

‘They hey must teach hospital staff that, here, it’s a place where we work with these kinds of people … Because some people [*staff*] here don’t know why they are here … This kind of hospital doesn’t need people like that.’ (Hloni, 48 years old, C4 incomplete)

### Real-life rehabilitation environment or home trials prior to discharge

Secondly, 54.5% (*n* = 6) of participants identified a need for the hospital environment to more closely resemble their home environments, or for regular access to realistic settings either within the hospital or through weekend home visits:

‘But if my space is not the same at home as here, then I can’t compare the two … ’ (John, 68 years old, L3 incomplete)

‘It is different. Look, this has spoiled me! So I’ve gone onto my bed, which is a king-size bed. I can’t do what I do here. My daughter has got rails, but I have to go in a single bed. I said I wanna sleep in my bed, but I don’t think I can. Maybe, eventually. But there’s nothing to grip.’ (Jane, 66 years old, T9 incomplete)

‘So the only difference is now, my wheelchair doesn’t fit in the bathroom.’ (Amy, 56 years old, T8 incomplete)

Weekend home trials were especially helpful because they provided realistic opportunities to practise skills in familiar physical environments. These visits also allowed participants and their families to identify and address environmental challenges before discharge, although some found their initial visits home challenging as a result of these differences:

‘You never expect a hospital ward to be something that warms you up for home. So the warm up for home is that I’ve been able to go home every weekend for a couple of days.’ (Mark, 48 years old, C1 incomplete)

‘Last weekend, I spent with the carer at my home. It was good. A few changes that needs to be done. We’ve seen it, we’ve looked at it. Now we gonna sort that out once I go back.’ (Jane, 66 years old, T9 complete)

### Enhancing psychological support

Thirdly, 45.5% (*n* = 5) of participants identified a need for more structured, formal and individualised psychological support. Only two participants planned to continue psychological intervention following discharge. Many expressed that the psychological support that they received during rehabilitation lacked structure, consistency and personalisation, leaving them feeling insufficiently prepared emotionally for their return home:

‘So if it [*psychology*] was just a little bit more specific on what I can expect and what I can control when I get here [home], then it would have been better.’ (Joe, 25 years old, C4 incomplete)

‘Can’t have sitting down and, “Ah, we gonna have a heart to heart,” at random times, random days … I mean, the psychologist came and sat down here the other day at 8 o’clock in the evening. I hadn’t seen them for 2 weeks … I think it’s an important role. But I mean if my experience was something to go by, I would hate to know somebody who needs it, how they’re gonna react to that.’ (Mark, 48 years old, C1 incomplete)

## Discussion

Our study aimed to explore the experiences of inpatient rehabilitation among people with SCI, how this affects their transition out of rehabilitation, and to identify needs to assist in this process. This is the first study to explore the experience of home transition for people with SCI in South Africa. Three themes emerged: (1) overall rehabilitation experience, (2) factors influencing rehabilitation experience and their influence on readiness to transition out of rehab and (3) needs of people with SCI when transitioning out of inpatient rehabilitation. The findings of our study show that, while most participants generally found their time in rehabilitation to be beneficial and positive, several factors shaped their experiences. This result was in alignment with previous studies (Mothabeng et al. 2007; Samuel et al. 2007). Key areas for improvement were identified, highlighting opportunities to bridge the gaps within the rehabilitation process, as well as in the transition out of rehabilitation to home.

The approach to care was a key factor influencing the rehabilitation experience, particularly in relation to staff attitudes and knowledge, communication and participants’ involvement in decision-making. The attitudes and expertise of healthcare workers play a crucial role in shaping patients’ experiences and readiness for transition (Conti et al. 2020; Moran, Barclay & Lannin 2022; Pryor, Haylen & Fisher 2021). In our study, while most healthcare professionals were described as helpful, friendly, and knowledgeable, some demonstrated gaps in attitude, knowledge, or skills, which negatively affected participants. Previous research supports this observation, showing that staff attitudes impact patients’ ability to achieve self-management goals and their sense of control (Bourke et al. 2014).

Additionally, communication and shared decision-making about their care impacted participants’ experiences.

Some patients felt excluded from decision-making about discharge plans, medical care, and scheduling, highlighting the need for greater autonomy and involvement. Patients with SCI often experience a diminished capacity to engage in decision-making during the early stages of rehabilitation as a result of physical, psychological, and environmental factors (Scheel-Sailer et al. 2017). This finding emphasises the importance of receiving adequate, personalised information to regain their decision-making capacity.

A patient-centred approach was one of the recommendations that participants made to assist patients’ transition from inpatient rehabilitation to home. A patient-centred approach prioritises listening to patients, incorporating their preferences, empowering individuals in their recovery and promoting self-management (Bourke et al. 2014; Lindberg et al. 2013).

The participants with SCI recommended three essential elements: the need for specialised SCI care, meaningful involvement in decision-making, and positive staff attitudes. Specifically, some participants identified a critical gap between skills learnt in structured therapy sessions and opportunities to practise these skills in the ward. This recognition, combined with feeling excluded from decision-making processes regarding their medical care, resulted in insufficient preparation for discharge, particularly concerning essential self-management skills such as bowel and bladder management. Effective communication, provision of information, and fostering an open atmosphere are essential for patient involvement (Melin et al. 2018). When nursing staff engage with patients on a personal level, they become more attuned to the patients’ situations, fostering closer relationships and improving the overall rehabilitation experience for patients with SCI (Steensgaard, Kolbaek & Angel 2022).

Specialised SCI care also extends beyond technical knowledge; such care includes ensuring that all rehabilitation staff clearly understand their roles and possess the skills to sensitively engage with patients undergoing profound life changes. Ongoing professional development programmes are therefore recommended, with particular attention to patient-centred care, self-management strategies, and effective communication (Krysa et al. 2022). Training initiatives focused on enhancing staff communication skills and promoting shared decision-making can directly address these identified gaps, ultimately fostering a supportive rehabilitation environment that prioritises patient autonomy and preparedness (Bourke et al. 2014; Lindberg et al. 2013). In low- and middle-income countries like South Africa, where resources are often constrained, cost-effective strategies such as modular training programmes, mentorship programmes, and integration of patient-centred care principles into existing national rehabilitation policies can help bridge these gaps. Practical steps include regular in-service training, incorporating patient-centred care modules into undergraduate and postgraduate health professional curricula, and developing locally relevant guidelines and checklists to standardise care practices within the appropriate context. Even within busy, under-resourced settings, regular, structured training for all staff can ensure that the priorities are patient autonomy, shared decision-making, and holistic preparedness for life after discharge.

The physical environment played a significant role in shaping the rehabilitation experience, consistent with findings from previous studies (Bourke et al. 2014; Colley, Zeeman & Kendall 2018; Nunnerley & Dean 2013; Simpson, Villeneuve & Clifton 2020). While participants generally appreciated the ward’s space and cleanliness, some found the main ward noisy and noted that bathroom equipment was occasionally unavailable or not in working order. The most notable concern was that the rehabilitation environment did not closely simulate their home settings, making it difficult to fully prepare for the transition. Many participants felt adequately prepared only after completing home visits or weekend trials. Previous research emphasises the importance of rehabilitation spaces that mirror real-life environments to facilitate a smoother transition to home (Bourke et al. 2014; Moran et al. 2022; Nunnerley & Dean 2013). While hospital safety and accessibility are essential, integrating elements that reflect the actual living conditions of the patients can better prepare them for home trials and promote greater independence upon discharge. However, implementing this approach may be challenging in the South African context, particularly in the public health sector, because of significant disparities in housing conditions across socio-economic groups (Mathee et al. 2021). Rehabilitation hospitals must consider adapting their hospital environment to more closely simulate real home environments to facilitate recovery and prepare for home transitions.

Being among other individuals with SCI during rehabilitation fostered belonging and reduced feelings of isolation. Participants described how peers with SCI offered encouragement and provided perspective by demonstrating that independence was possible, supporting findings by Machida, Irwin and Feltz (2013). These findings also align with research showing that peer connection supports adjustment by offering hope, normalising experiences, and reducing loneliness (Sweet et al. 2021). However, some participants anticipated difficulty adjusting once this natural peer network was no longer present at home, highlighting the value of structured peer mentorship to extend these benefits beyond hospital discharge (Sweet et al. 2021).

Similar to previous studies, patients’ mental health can affect the rehabilitation experience and outcomes (Mothabeng et al. 2007). Psychosocial factors such as patients’ emotions, dependence on health professionals, and insight into health conditions play a large part in the rehabilitation experience (Samuel et al. 2007).

Although those studies were conducted almost 2 decades ago, our current study echoes their findings. People with SCI, including those in our present study, experience trauma, confusion, grief, and distress in the acute and subacute phases following their injuries (Bender & Burgess 2020; Bourke et al. 2014; Clifton 2014; Joseph et al. 2016; Machida et al. 2013; Nunnerley & Dean 2013).

While participants in our present study felt encouraged and supported by various staff members at the rehabilitation hospital, some did not feel that their psychological needs were adequately met. Many felt that this support lacked structure, individualisation, and consistency during rehabilitation. In addition, some participants felt that, while they were physically prepared for discharge, they were not psychologically prepared for returning home or to the community with their disabilities. Many participants planned to continue with physical rehabilitation after discharge, but only two expressed an intention to seek psychological support. Bourke et al. (2014) also highlight this gap in psychological support around navigating community members’ attitudes. Some participants expressed mixed feelings about discharge: they were eager to return home, but apprehensive about reintegrating into their communities. Mpho’s concern about the attitudes towards him in the township reflects the anticipated stigma or judgement based on his disability. Recent research shows that stigma after SCI often persists beyond the hospital stay and can significantly impact QoL and social participation (Budd, Gater & Channell 2022; Ownsworth et al. 2024). In southern Africa, a study from Namibia found that lack of community acceptance was a source of distress for individuals with SCI (Ashipala & Langendorf 2022). These findings highlight that successful community reintegration depends on more than physical readiness. It requires meaningful social acceptance and opportunities for participation. This conclusion aligns with global recommendations, which advocate for interventions targeting both individual coping strategies and broader societal attitudes (Budd et al. 2022). Addressing stigma within rehabilitation planning may therefore be critical to improving long-term outcomes for individuals with SCI in South Africa.

The psychological state of the person with SCI can have an impact on their choices to participate in social or community activities (Joseph et al. 2016). Clifton (2014:1825), in his autoethnographic paper following his own SCI, describes SCI as a ‘body/mind separation’ and a deeply personal loss. Joseph et al. (2016:1376) also found that people with SCI needed to learn to deal with their ‘new self’. This skill requires psychological interventions which help people with SCI rebuild their personal and new life narratives (Clifton 2014). These findings underscore the critical need for a structured, formalised, and individualised approach to psychological support, both during rehabilitation and beyond, to better equip people with SCI for their transition and long-term well-being.

The findings of our study highlight possible gaps in SCI rehabilitation in South Africa, which mirror the systemic challenges, including limited access to specialised services, inadequately trained staff, and weak referral systems (Louw et al. 2023). As South Africa moves towards the implementation of the NHI, there is an opportunity to address these issues and bolster rehabilitation care for people with SCI. Integrating specialised SCI rehabilitation into the NHI framework could help standardise care and ensure that all patients have access to comprehensive services. Priority actions include developing national rehabilitation guidelines, strengthening referral pathways, and implementing systems to monitor outcomes. Workforce reinforcement is also essential, focusing on recruiting and retaining skilled rehabilitation professionals. Aligning these strategies with NHI reforms could promote successful community reintegration and a better QoL for people with SCI.

### Limitations

Our study has several limitations. It was conducted at a single, privately funded rehabilitation hospital, limiting the generalisability and transferability of the findings, particularly to public healthcare settings. Our study was conducted in an urban area, thus limiting the transferability of findings to participants living in rural areas.

Purposive sampling may have introduced responder bias. Most participants were men with traumatic, incomplete, and thoracic-level SCI, restricting the applicability of results to those with different injury profiles, including those with higher-level lesions, who have different functional and participation challenges. In addition, there may have been translation bias impacting the meaning in the English translations of the Zulu interviews. In South Africa, specialised SCI rehabilitation is largely limited to privately funded, urban hospitals, yet most of the population lacks private health insurance and relies on under-resourced public facilities (Joseph et al. 2017; Mayosi & Benatar 2014). These disparities may significantly affect the rehabilitation experience. Future research should include both private and public healthcare settings to better inform system-level improvements.

## Conclusion

Our study provided valuable insights into the rehabilitation experiences of people with SCI in South Africa and identified critical needs impacting their readiness to transition home, including a specialised and patient-centred approach to care (including staff attitudes and knowledge, communication, and involvement in decision-making), bridging the gap between hospital and home physical environments, and more structured and individualised psychological support. With no contextual SCI model of care, future research across diverse healthcare settings is required to inform structured rehabilitation systems in favour of promoting QoL, reintegration, and participation upon discharge.

## Appendix 1

**TABLE 1-A1:** The coding process.

Step	Description	Example (as applicable)
Familiarisation with transcripts	Reading and re-reading transcripts to gain clear understanding of the data, as well as making notes on initial impressions on transcripts	‘Positive experience of rehab’ ‘Rehab is helpful’ ‘Patient’s decision-making regarding BBM not taken seriously’
Initial coding	Assigning descriptive codes to meaningful segments of data, inductively (from participants’ narrative) and deductively (using ICF framework and interview guides from similar papers)	‘Staff skill/knowledge’ ‘Individuality’ ‘Psychology’
Grouping codes	Organising similar codes into broader categories	‘Physical environment’ ‘Care approach’ ‘Emotions’ ‘Perceived readiness to go home’
Developing themes	Refining themes into greater themes that answer the objectives	‘Overall experience’ ‘Factors influencing experience’ ‘Needs’
Reviewing themes	Checking themes against the data to ensure accuracy, re-reading transcripts	-
Finalising and naming themes	Final theme names developed collaboratively	-

**TABLE 1-A2a:** Distribution of participant responses across themes and subthemes identified in the qualitative analysis (*N* = 11).

Participant code	Overall experience			
	Positive	Negative	Mixed	All hospital services received
P1	1.0	0.0	0.0	1.0
P2	1.0	0.0	0.0	1.0
P3	1.0	0.0	0.0	1.0
P4	1.0	0.0	0.0	1.0
P5	0.0	0.0	1.0	1.0
P6	1.0	0.0	0.0	0.0
P7	1.0	0.0	0.0	1.0
P8	0.0	0.0	1.0	1.0
P9	0.0	0.0	1.0	1.0
P10	1.0	0.0	0.0	0.0
P11	1.0	0.0	0.0	1.0
*n* =	8.0	0.0	3.0	9.0
%	72.7	0.0	27.2	81.8

**TABLE 1-A2b:** Distribution of participant responses across themes and subthemes identified in the qualitative analysis (*N* = 11).

Influencing factors								
Environmental factors								
Care approach (staff attitudes, communication, involvement in decision-making)			Physical environment			Peer support		
Positive	Negative	Mixed	Positive	Negative	Mixed	Positive	Negative	Mixed
0.0	0.0	1.0	0.0	0.0	0.0	1.0	0.0	0.0
0.0	0.0	1.0	0.0	0.0	0.0	1.0	0.0	0.0
1.0	0.0	0.0	0.0	0.0	1.0	1.0	0.0	0.0
1.0	0.0	0.0	0.0	0.0	1.0	0.0	0.0	0.0
0.0	1.0	0.0	0.0	0.0	1.0	0.0	0.0	0.0
0.0	0.0	1.0	1.0	0.0	0.0	0.0	0.0	0.0
1.0	0.0	0.0	0.0	0.0	1.0	1.0	0.0	0.0
0.0	0.0	1.0	0.0	0.0	1.0	1.0	0.0	0.0
0.0	0.0	1.0	0.0	0.0	1.0	0.0	0.0	1.0
0.0	0.0	1.0	0.0	0.0	1.0	0.0	0.0	0.0
0.0	0.0	1.0	0.0	0.0	1.0	0.0	0.0	1.0
3.0	1.0	7.0	1.0	0.0	8.0	5.0	0.0	2.0
27.2	9.1	63.6	9.1	0.0	72.7	45.5	0.0	18.2

**TABLE 1 A-1Ab:** Distribution of participant responses across themes and subthemes identified in the qualitative analysis (*N* = 11).

Influencing factors				
Personal factors				
Emotions	Beliefs and motivation	Perceived readiness for discharge		
		Positive	Negative	Mixed
1.0	0.0	1.0	0.0	0.0
1.0	1.0	0.0	0.0	1.0
1.0	1.0	0.0	0.0	0.0
1.0	1.0	1.0	0.0	0.0
1.0	0.0	0.0	1.0	0.0
1.0	1.0	N/A	N/A	N/A
0.0	0.0	1.0	0.0	0.0
1.0	1.0	0.0	0.0	1.0
1.0	1.0	1.0	0.0	0.0
1.0	1.0	1.0	0.0	0.0
1.0	1.0	1.0	0.0	0.0
10.0	8.0	6.0	1.0	2.0
90.1	72.7	54.5	9.1	18.2

**TABLE 1-A2c:** Distribution of participant responses across themes and subthemes identified in the qualitative analysis (*N* = 11).

Needs		
Patient-centred approach	Real-life rehab environment and/or value of home trials	Enhanced psychological support
0.0	0.0	1.0
1.0	0.0	0.0
0.0	0.0	1.0
0.0	0.0	0.0
1.0	1.0	1.0
0.0	1.0	0.0
0.0	1.0	0.0
1.0	1.0	1.0
0.0	1.0	0.0
1.0	0.0	0.0
1.0	1.0	1.0
5.0	6.0	5.0
45.5	54.5	45.5

## References

[CIT0001] Ashipala, D.O. & Langendorf, L., 2022, ‘Experiences of spinal cord injury patients admitted to the rehabilitation unit at the national referral hospital in Khomas Region, Namibia’, *African Journal of Disability* 11, a1018. 10.4102/ajod.v11i0.1018 PMC9350479 35936925

[CIT0002] Bender, A.A. & Burgess, E.O., 2020, ‘Constructing recovery narratives: Experiences and expectations following spinal cord injury’, *Rehabilitation Nursing* 45(5), 254–262. 10.1097/RNJ.0000000000000202 32865946

[CIT0003] Bourke, J.A., Hay-Smith, E.J.C., Snell, D.L. & DeJong, G., 2014, ‘Attending to biographical disruption: The experience of rehabilitation following tetraplegia due to spinal cord injury’, *Disability and Rehabilitation* 37(4), 296–303. 10.3109/09638288.2014.918188 24828314

[CIT0004] Budd, M.A., Gater, D.R., Jr. & Channell, I., 2022, ‘Psychosocial consequences of spinal cord injury: A narrative review’, *Journal of Personalized Medicine* 12(7), 1178. 10.3390/jpm12071178 35887675 PMC9320050

[CIT0005] Cheng, C.L., Plashkes, T., Shen, T., Fallah, N., Humphreys, S., O’Connell, C. et al., 2017, ‘Does specialized inpatient rehabilitation affect whether or not people with traumatic spinal cord injury return home?’ *Journal of Neurotrauma* 34(20), 2867–2876. 10.1089/neu.2016.4930 28447870

[CIT0006] Clifton, S., 2014, ‘Grieving my broken body: An autoethnographic account of spinal cord injury as an experience of grief’, *Disability and Rehabilitation* 36(21), 1823–1829. 10.3109/09638288.2013.872202 24382269

[CIT0007] Colley, J., Zeeman, H. & Kendall, E., 2018, ‘“Everything happens in the hallways”: Exploring user activity in the corridors at two rehabilitation units’, *Health Environments Research and Design Journal* 11(2), 163–176. 10.1177/1937586717733149 29069918

[CIT0008] Conti, C., Dimonte, V., Rizzi, A., Clari, M., Mozzone, S., Garrino, L. et al., 2020, ‘Barriers and facilitators of education provided during rehabilitation of people with spinal cord injuries: A qualitative description’, *PLoS One* 15(10), 1–14. 10.1371/journal.pone.0240600 PMC7561131 33057362

[CIT0009] Creswell, J.W., 1998, *Qualitative inquiry and research design: Choosing among five traditions*, Sage, Thousand Oaks, CA.

[CIT0010] Du Plessis, M., McGaffin, C.R., Molepo, T., Oelofse, R., Van Zyl, S. & Mashola, M.K., 2018, ‘Perceived readiness for hospital discharge: Patients with spinal cord injury versus physiotherapists’, *South African Journal of Physiotherapy* 74(1), 437. 10.4102/sajp.v74i1.437 30167501 PMC6111384

[CIT0011] Guest, G., Bunce, A. & Johnson, L., 2006, ‘How many interviews are enough?’ *Field Methods* 18, 59–82. 10.1177/1525822X05279903

[CIT0012] Ho, C., Atchison, K., Noonan, V.K., McKenzie, N., Cadel, L., Ganshorn, H. et al., 2021, ‘Models of care delivery from rehabilitation to community for spinal cord injury: A scoping review’, *Journal of Neurotrauma* 38(6), 677–697. 10.1089/neu.2020.7396 33191849

[CIT0013] Joseph, C., Ernst, S., Virginia, W., Joyce, M. & Francois, T., 2017, ‘People with spinal cord injury in Republic of South Africa’, *American Journal of Physical Medicine and Rehabilitation* 96(2), S109–S111. 10.1097/PHM.0000000000000594 28059894

[CIT0014] Joseph, C., Wahman, K., Phillips, J. & Wikmar, L.N., 2016, ‘Client perspectives on reclaiming participation after a traumatic spinal cord injury in South Africa’, *Physical Therapy* 96(9), 1372–1380. 10.2522/ptj.20150258 27081200

[CIT0015] Kashif, M., Jones, S., Darain, H., Iram, H., Raqib, A. & Butt, A.A., 2019, ‘Factors influencing the community integration of patients following traumatic spinal cord injury: A systematic review’, *Journal of the Pakistan Medical Association* 69(9), 1336–1342. 31511721

[CIT0016] Krefting, L., 1991, ‘Rigor in qualitative research: The assessment of trustworthiness’, *American Journal of Occupational Therapy* 45(3), 214–222. 10.5014/ajot.45.3.214 2031523

[CIT0017] Krysa, J.A., Gregorio, M.P., Pohar Manhas, K., MacIsaac, R., Papathanassoglou, E. & Ho, C.H., 2022, ‘Empowerment, communication, and navigating care: The experience of persons with spinal cord injury from acute hospitalization to inpatient rehabilitation’, *Frontiers in Rehabilitation Sciences* 3, 1–11. 10.3389/fresc.2022.904716 PMC9397833 36188987

[CIT0018] Lindberg, J., Kreuter, M., Taft, C. & Person, L.-O., 2013, ‘Patient participation in care and rehabilitation from the perspective of patients with spinal cord injury’, *Spinal Cord* 51, 834–837. 10.1038/sc.2013.97 23999110

[CIT0019] Louw, Q.A., Conradie, T., Xuma-Soyizwapi, N., Davis-Ferguson, M., White, J., Stols, M. et al., 2023, ‘Rehabilitation capacity in South Africa – A situational analysis’, *International Journal of Environmental Research and Public Health* 20, 3579. 10.3390/ijerph20043579 36834271 PMC9961618

[CIT0020] Machida, M., Irwin, B. & Feltz, D., 2013, ‘Resilience in competitive athletes with spinal cord injury: The role of sport participation’, *Qualitative Health Research* 23(8), 1054–1065. 10.1177/1049732313493673 23771633

[CIT0021] Madasa, V., Boggenpoel, B., Phillips, J. & Joseph, C., 2020, ‘Mortality and secondary complications four years after traumatic spinal cord injury in Cape Town, South Africa’, *Spinal Cord Series and Cases* 6(1), 84. 10.1038/s41394-020-00334-w 32887870 PMC7473856

[CIT0022] Mashola, M.K., Olorunju, S.A.S. & Mothabeng, J., 2019, ‘Factors related to hospital readmissions in people with spinal cord injury in South Africa’, *South African Medical Journal* 109(2), 107–111. 10.7196/SAMJ.2019.v109i2.13344 30834861

[CIT0023] Mathee, A., Moyes, J., Mkhencele, T., Kleynhans, J., Language, B., Piketh, S. et al., 2021, ‘Housing quality in a rural and an urban settlement in South Africa’, *International Journal of Environmental Research and Public Health* 18(5), 2240. 10.3390/ijerph18052240 33668301 PMC7956558

[CIT0024] Mayosi, B.M. & Benatar, S.R., 2014, ‘Health and health care in South Africa – 20 Years after Mandela’, *New England Journal of Medicine* 371(14), 1344–1353. 10.1056/NEJMsr1405012 25265493

[CIT0025] Melin, J., Persson, L.O., Taft, C. & Kreuter, M., 2018, ‘Patient participation from the perspective of staff members working in spinal cord injury rehabilitation’, *Spinal Cord* 56(6), 614–620. 10.1038/s41393-018-0061-7 29367656

[CIT0026] Moran, K., Barclay, L. & Lannin, N.A., 2022, ‘Experiences of people with non-traumatic spinal cord injuries returning home after inpatient rehabilitation’, *Disability and Rehabilitation* 46(2), 362–368. 10.1080/09638288.2022.2162611 36591728

[CIT0027] Morris, L.D., Grimmer, K.A., Twizeyemariya, A., Coetzee, M., Leibbrandt, D.C. & Louw, Q.A., 2021, ‘Health system challenges affecting rehabilitation services in South Africa’, *Disability and Rehabilitation* 43(6), 877–883. 10.1080/09638288.2019.1641851 31378096

[CIT0028] Morse, J.M., 1995, ‘The significance of saturation’, *Qualitative Health Research* 5(2), 147–149. 10.1177/104973239500500201

[CIT0029] Mothabeng, D., Malinga, C.P., Van der Merwe, C., Qhomane, P.T. & Motjotji, S.N., 2007, ‘The views of patients with spinal cord injuries on their rehabilitation experience’, *South African Journal of Physiotherapy* 63(3), 22–25. 10.4102/sajp.v63i3.139

[CIT0030] Nizeyimana, E., Louw, Q. & Joseph, C., 2025, ‘A community reintegration model for persons with traumatic spinal cord injury in South Africa: Process and outcomes’, *Advances in Rehabilitation Science and Practice* 14, 1–10. 10.1177/27536351251326797 PMC11967211 40182655

[CIT0031] Nowell, L.S., Norris, J.M., White, D.E. & Moules, N.J., 2017, ‘Thematic analysis: Striving to meet the trustworthiness criteria’, *International Journal of Qualitative Methods* 16(1), 1–13. 10.1177/1609406917733847

[CIT0032] Nunnerley, J.L. & Dean, S.G., 2013, ‘Leaving a spinal unit and returning to the wider community: An interpretative phenomenological analysis’, *Disability and Rehabilitation* 35(14), 1164–1173. 10.3109/09638288.2012.723789 23035858

[CIT0033] Ownsworth, T., Mols, H., O’Loghlen, J., Xie, Y., Kendall, M., Nielsen, M. et al., 2024, ‘Stigma following acquired brain injury and spinal cord injury: Relationship to psychological distress and community integration in the first-year post-discharge’, *Disability and Rehabilitation* 46(9), 1796–1806. 10.1080/09638288.2023.2205173 37128900

[CIT0034] Pefile, N., Mothabeng, J.D. & Naidoo, S., 2019, ‘Profile of patients with spinal cord injuries in Kwazulu-Natal, South Africa: Implications for vocational rehabilitation’, *Journal of Spinal Cord Medicine* 42(6), 709–718. 10.1080/10790268.2018.1428264 29388905 PMC6830293

[CIT0035] Pilusa, S., Myezwa, H. & Potterton, J., 2021, ‘Secondary health conditions in people with spinal cord injury in South Africa: Prevalence and associated factors’, *South African Medical Journal* 111(12), 1211–1217. 10.7196/SAMJ.2021.v111i12.15761 34949309

[CIT0036] Pryor, J., Haylen, D. & Fisher, M., 2021, ‘Problems people with spinal cord injury experience accessing help with bowel care when hospitalised outside a specialist spinal injury service’, *Journal of Clinical Nursing* 30(11–12), 1633–1644. 10.1111/jocn.15717 33590956

[CIT0037] Samuel, V.M., Moses, J., North, N., Smith, H. & Thorne, K., 2007, ‘Spinal cord injury rehabilitation: The experience of women’, *Spinal Cord* 45(12), 758–764. 10.1038/sj.sc.3102111 17768426

[CIT0038] Saunders, B., Sim, J., Kingstone, T., Baker, S., Waterfield, J., Bartlam, B. et al., 2018, ‘Saturation in qualitative research: Exploring its conceptualization and operationalization’, *Quality & Quantity* 52(4), 1893–1907. 10.1007/s11135-017-0574-8 29937585 PMC5993836

[CIT0039] Scheel-Sailer, A., Post, M.W., Michel, F., Weidmann-Hügle, T. & Hölzle, R.B., 2017, ‘Patients’ views on their decision making during inpatient rehabilitation after newly acquired spinal cord injury – A qualitative interview-based study’, *Health Expect* 20(5), 1133–1142. 10.1111/hex.12559 28338280 PMC5600242

[CIT0040] Simpson, B., Villeneuve, M. & Clifton, S., 2020, ‘The experience and perspective of people with spinal cord injury about well-being interventions: A systematic review of qualitative studies’, *Disability and Rehabilitation* 44(14), 3349–3363. 10.1080/09638288.2020.1864668 33377801

[CIT0041] Smith, E., Sakakibara, B. & Miller, W., 2014, ‘A review of factors influencing participation in social and community activities for wheelchair users’, *Disability and Rehabilitation: Assistive Technology* 11, 1–14. 10.3109/17483107.2014.989420 25472004 PMC4581875

[CIT0042] Steensgaard, R., Kolbaek, R. & Angel, S., 2022, ‘Nursing staff facilitate patient participation by championing the patient’s perspective: An action research study in spinal cord injury rehabilitation’, *Health Expectations* 25(5), 2525–2533. 10.1111/hex.13574 36004714 PMC9615065

[CIT0043] Sweet, S.N., Hennig, L., Shi, Z., Clarke, T., Flaro, H., Hawley, S. et al., 2021, ‘Outcomes of peer mentorship for people living with spinal cord injury: Perspectives from members of Canadian community-based SCI organizations’, *Spinal Cord* 59, 1301–1308. 10.1038/s41393-021-00725-2 34732859 PMC8565648

[CIT0044] Tsai, I.-H., Graves, D.E., Chan, W., Darkoh, C., Lee, M.-S. & Pompeii, L.A., 2017, ‘Environmental barriers and social participation in individuals with spinal cord injury’, *Rehabilitation Psychology* 62(1), 36–44. 10.1037/rep0000117 28045281 PMC5899919

[CIT0045] Turner-Stokes, L., Williams, H., Bill, A., Bassett, P. & Sephton, K., 2016, ‘Cost-efficiency of specialist inpatient rehabilitation for working-aged adults with complex neurological disabilities: A multicentre cohort analysis of a national clinical data Set’, *BMJ Open* 6(2), e010238. 10.1136/bmjopen-2015-010238 PMC4769383 26911586

[CIT0046] World Health Organization (WHO), 2002, *Towards a common language for functioning, disability and health: ICF*, WHO, Geneva, viewed 16 July 2021, from https://www.who.int/classifications/icf/icfbeginnersguide.%0Apdf?ua=1%0A.

[CIT0047] World Health Organization (WHO), 2017, *Rehabilitation 2030: A call for action*, WHO, Geneva, viewed 16 July 2021, from https://www.who.int/publications/i/item/rehabilitation-2030-a-call-for-action.

